# Neural anomalies during vigilance in schizophrenia: Diagnostic specificity and genetic associations

**DOI:** 10.1016/j.nicl.2020.102414

**Published:** 2020-09-08

**Authors:** Samuel D. Klein, Laurie L. Shekels, Kathryn A. McGuire, Scott R. Sponheim

**Affiliations:** a University of Minnesota Clinical Science and Psychopathology Research Program, University of Minnesota-Twin Cities, 75 East River Road, Minneapolis, MN 55455, USA; b Minneapolis Veterans Affairs Health Care System, 1 Veterans Dr. Minneapolis, MN 55417, USA; c University of Minnesota, Department of Psychiatry and Behavioral Science, 606 24th Ave S, Minneapolis, MN 55454, USA

**Keywords:** DS-CPT, Degraded stimulus continuous performance task, COMT, Catechol-O-methyltransferase, ERPs, Event-related potential, VA, Veterans Affairs, SZ, Patients with schizophrenia, BP, Patients with bipolar disorder, HC, Healthy Controls, SZRel, First-degree relatives of patient with schizophrenia, BPRel, First-degree relatives of patient with bipolar disorder, FDR, False discovery rate, Schizophrenia, Bipolar disorder, Vigilance, Electrophysiology, Genetic liability, Evoked potential, Endophenotype

## Abstract

Impaired vigilance is a core cognitive deficit in schizophrenia and may serve as an endophenotype (i.e., mark genetic liability). We used a continuous performance task with perceptually degraded stimuli in schizophrenia patients (*N = 48)*, bipolar disorder patients (*N = 26)*, first-degree biological relatives of schizophrenia patients (*N* = 55) and bipolar disorder patients (*N* = 28), as well as healthy controls (*N* = 68) to clarify whether previously reported vigilance deficits and abnormal neural functions were indicative of genetic liability for schizophrenia as opposed to a generalized liability for severe psychopathology. We also examined variation in the Catechol-O-methyltransferase gene to evaluate whether brain responses were related to genetic variation associated with higher-order cognition. Relatives of schizophrenia patients had an increased rate of misidentification of nontarget stimuli as targets when they were perceptually similar, suggestive of difficulties with contour perception. Larger early visual responses (i.e., N1) were associated with better task performance in patients with schizophrenia consistent with enhanced N1 responses reflecting beneficial neural compensation. Additionally, reduced N2 augmentation to target stimuli was specific to schizophrenia. Both patients with schizophrenia and first-degree relatives displayed reduced late cognitive responses (P3b) that predicted worse performance. First-degree relatives of bipolar patients exhibited performance deficits, and displayed aberrant neural responses that were milder than individuals with liability for schizophrenia and dependent on sex. Variation in the Catechol-O-methyltransferase gene was differentially associated with P3b in schizophrenia and bipolar groups. Poor vigilance in schizophrenia is specifically predicted by a failure to enhance early visual responses, weak augmentation of mid-latency brain responses to targets, and limited engagement of late cognitive responses that may be tied to genetic variation associated with prefrontal dopaminergic availability. Experimental results illustrate specific neural functions that distinguish schizophrenia from bipolar disorder and provides evidence for a putative endophenotype that differentiates genetic liability for schizophrenia from severe mental illness more broadly.

## Highlights

• Compared visual ERPs in schizophrenia probands, bipolar probands, and relatives.
• Enhanced N1 in patients with schizophrenia predicted greater perceptual sensitivity.
• A schizophrenia specific N2 deficit may reflect impaired visual object recognition.
• Schizophrenia probands and relatives displayed reduced P3 varying by COMT genotype.
• Bipolar probands and relatives had milder abnormalities dependent on sex.

## 1 Introduction

Impaired vigilance represents a core cognitive deficit in schizophrenia, with evidence suggesting that it may mark genetic liability for the disorder (i.e., be an endophenotype; Cornblatt and Malhotra, 2001, Gottesman and Gould, 2003, Green et al., 2004). Patients with schizophrenia generally demonstrate poor vigilance on sustained attention tasks, with some studies reporting similarly reduced performance in first-degree biological relatives (Chen and Faraone, 2000, Demeter et al., 2013, Nestor et al., 1990, Wohlberg and Kornetsky, 1973). The degraded stimulus continuous performance task (DS-CPT) reliably yields a linear decline in performance over the task’s duration indicative of measures of sustained visual attention (Nuechterlein et al., 1983, Nuechterlein, 1991, Parasuraman, 1979). Given that patients with schizophrenia and their biological relatives have failed to demonstrate steeper linear declines in performance compared to healthy controls—they exhibit a consistent degree of impairment in perceptual sensitivity (dˊ) across the duration of the task (Nuechterlein, 1983, Sponheim et al., 2006)—impaired performance may reflect underlying abnormalities in visual perception. To examine neural contributions to visual perception as tapped by the DS-CPT and to test the specificity of abnormalities to genetic liability for schizophrenia, we carried out an analysis of event-related potentials to DS-CPT stimuli in patients with schizophrenia, patients with bipolar disorder, and first-degree biological relatives of both patient types.

Previous reports have documented that genetic liability for schizophrenia may be related to reduced sensitivity on CPTs that use visually degraded stimuli. In studies that employed CPTs with degraded stimuli, patients with schizophrenia had lower dˊ values relative to healthy controls (Liu et al., 1997, Nuechterlein et al., 2015), with similar deficits in perceptual sensitivity observed in first-degree relatives (Asarnow et al., 2002, Grove et al., 1991, Maier et al., 1992). Critically, the reduced sensitivity for differentiation of target stimuli from nontargets (i.e. vigilance) is not due to difficulty sustaining attention over the duration of the task since the size of the deficit is comparable at the beginning and ending blocks of trials (Buchanan et al., 1997; Fleck et al., 2001, Knott et al., 1999, Nestor et al., 1990, Sponheim et al., 2006). It may be that failures in perceptual processes required to visually discriminate degraded task stimuli are the source of impaired vigilance on the DS-CPT.

The neural correlates of reduced vigilance can be evaluated using the event-related potential (ERP) technique. Koelega et al. found that vigilance may be related to electrophysiological responses within the 250–650 msec after stimulus presentation (Koelega et al., 1992). In tasks tapping visual attention, a decrement in P3 components has been consistently demonstrated in schizophrenia (Martínez et al., 2012, Potts et al., 2002, VanMeerten et al., 2016, Wood et al., 2007). Specific to the DS-CPT, Knott et al. found patients with schizophrenia had a diminution in P3 amplitudes relative to healthy controls (Knott et al., 1999). Previously, we reported that early sensory responses (N1 components) were augmented in first-degree relatives by vigilance demands of the DS-CPT, but not in patients, and that later neural responses (e.g., P300) were tied to target detection and were reduced in both patients and relatives (Sponheim et al., 2006). Thus, ERPs elicited by the DS-CPT may reflect neural functions associated with genetic liability for schizophrenia (i.e., be an endophenotype).

Both schizophrenia and bipolar disorder have been associated with abnormal perceptual functions measured with ERPs. Early auditory processing deficits demonstrated by reductions in early neural response (i.e. N1) have been well documented in schizophrenia (Ford et al., 2010, Foxe et al., 2011, Salisbury et al., 2010), with evidence suggesting such a deficit differentiates patients with schizophrenia and first-degree relatives from patients with bipolar disorder and first-degree relatives (Force et al., 2008). In addition, deficient auditory ERPs reflective of mid-latency processing (i.e. N2) also appear to distinguish schizophrenia from bipolar disorder (O’Donnell et al., 2004), and may distinguish patients with schizophrenia from their first-degree relatives (i.e. reflect a disorder specific deficit; Force et al., 2008). Later cognitive functions embodied in the P3 have been demonstrated in both patients with schizophrenia (Ford, 1999, Jeon and Polich, 2003) and bipolar disorder (O’Donnell et al., 2004), though other studies have reported amplitude reductions differentiate schizophrenia from bipolar disorder (Salisbury et al., 1998). It appears that abnormal auditory P3 may be a marker of functional psychosis rather than being specific to schizophrenia (Bestelmeyer et al., 2009, Hall et al., 2009, Salisbury et al., 1999). ERPs in response to visual stimuli in the context of the two disorders have been relatively understudied. However, researchers have found visual N1 amplitudes to be reduced in patients with schizophrenia (Neuhaus et al., 2011a), and enlarged in first-degree relatives of patients with schizophrenia and first-degree relatives of patients with bipolar disorder (VanMeerten et al., 2016). Deficits in visual N2 have been reported in schizophrenia (Ford et al., 1994, Potts et al., 2002) as well as bipolar disorder (Maekawa et al., 2013). To our knowledge, the visual N2 has never been directly compared in the two patient groups. Finally, patients with schizophrenia appear to have reduced P3 in response to visual stimuli (Ergen et al., 2008, Neuhaus et al., 2011b, Pfefferbaum et al., 1989) which appears to distinguish the neuropathology of schizophrenia from neural phenomena in major depression (Neuhaus et al., 2010) and bipolar disorder (Bestelmeyer, 2012). An analysis of neural responses to visual stimuli that involves affected individuals with schizophrenia and bipolar disorder and unaffected first-degree relatives of individuals with each disorder has not been reported. Such a comparison would shed light on the nature of the neurobiological deficits related to disease expression and genetic liability for the two disorders.

Establishing which manifestations of psychopathology relate to an abnormality is central to establishing an endophenotype. The DS-CPT has been employed in a number of clinical populations, with evidence that the task yields specific diagnostic effects. For instance, euthymic bipolar disorder patients performed similarly to healthy controls while manic patients performed more poorly, suggesting mania impairs vigilance (Fleck et al., 2005). Given the shared genetic overlap between bipolar disorder and schizophrenia (Purcell et al., 2009) a number of studies have employed the DS-CPT in both patient types to determine if impaired vigilance is shared across forms of severe psychopathology. Results suggest that schizophrenia is differentiated from bipolar disorder by reduced perceptual sensitivity (Kumar et al., 2010; S. K. Liu et al., 2002) and longer RTs to targets (Fleck et al., 2001). Likewise, reduced perceptual sensitivity on the DS-CPT distinguished individuals with a psychotic disorder from those with major depressive disorder (Nelson et al., 1998). Thus, reduced perceptual sensitivity on the DS-CPT appears to differentiate schizophrenia from other forms of severe mental illness.

Identifying specific elements of genetic variation associated with a candidate endophenotype may help clarify the genetic and neurobiological substrates of the disorder (Greenwood et al., 2012). Several investigations have linked deletions of chromosome 22q11 to enhanced susceptibility of schizophrenia (Bassett et al., 1998, Bassett et al., 1998, Bassett et al., 2003; M. Karayiorgou et al., 2010; M. Karayiorgou et al., 1995). One specific deletion occurs within the region coding for the Catechol-O-methyltransferase gene (COMT; Brisch et al., 2009). A number of studies have related the Val^158^Met COMT single nucleotide polymorphism (SNP) to both schizophrenia and poor performance on cognitive tasks associated with frontal lobe function (Egan et al., 2001, Fan et al., 2005, Malhotra et al., 2002, Winterer et al., 2006). Critically, COMT polymorphisms implicated in schizophrenia are also associated with bipolar disorder (Shifman et al., 2004, Zhang et al., 2009), and both disorders are characterized by impairments in higher-order cognition (Green, 2006, Morice, 1990, Vöhringer et al., 2013). COMT polymorphisms are also related to self-reported anhedonia in relatives of patients with schizophrenia, but not relatives of bipolar disorder patients (Docherty and Sponheim, 2008) suggesting differences in genetic liability for the two disorders. The COMT Val allele has been associated with positive symptoms in schizophrenia, while Met homozygosity is associated with positive symptoms in bipolar disorder (Goghari and Sponheim, 2008) also supporting a differential role in the clinical expression of the disorder.

Nonetheless, evidence for a direct association between a diagnosis of schizophrenia and COMT variation is mixed: a number of *meta*-analyses have failed to find any such association (Munafò et al., 2005, Okochi et al., 2009, Taylor, 2018), while others demonstrate a clear association (Costas et al., 2011, González-Castro et al., 2016, Williams et al., 2007). Additionally, the link between cognition and COMT remains equivocal. A 2008 *meta*-analysis by Barnett and colleagues suggests there is a robust though small (i.e. d = 0.06) pooled effect between COMT genotype and IQ. However, in the same *meta*-analysis the effect of COMT genotype on cognitive control (N-back task performance) was significantly larger for patient groups (d = 0.40) than non-patient groups (d = .-27, p < .05) though the authors found evidence of publication bias. A more recent *meta*-analysis found no link between IQ and COMT genotype (Geller et al., 2017). Critical to the present study, a *meta*-analytic review of neuroimaging studies revealed a robust link between activation in frontal regions and executive function paradigms (d = 0.73) favoring individuals with Met alleles over Val (Mier et al., 2010), with no evidence of publication bias. Additionally, there is strong evidence that COMT influences dopaminergic tone in prefrontal cortex, thereby modulating the effect of dopaminergic drugs (Schacht, 2016), and hypodopaminergia in frontal cortex has been associated with potentiated N2 (Rangel-Gomez et al., 2013) and P3 component amplitudes (Polich and Criado, 2006).

In order to clarify neural abnormalities associated with vigilance deficits in schizophrenia, and to evaluate the diagnostic specificity of abnormal brain responses elicited by the DS-CPT, we included schizophrenia patients, bipolar patients, and first degree-relatives of each patient group in the current analysis. We examined SNPs of the COMT gene to evaluate whether brain responses elicited by the DS-CPT may be specifically associated with a select aspect of genetic variation that has been related to both schizophrenia and bipolar disorder (Docherty and Sponheim, 2008, Goghari and Sponheim, 2008, Silberschmidt and Sponheim, 2008, Venables et al., 2009). The present work was designed to address three specific questions: 1) Do augmented early (i.e. N1) potentials indicative of visual processing differentiate genetic liability for schizophrenia from genetic liability for bipolar disorder?; 2) Do patients with bipolar disorder and their first-degree relatives share aberrant middle (i.e. N2) and late (i.e. P3b) latency posterior brain potentials seen in patients with schizophrenia and their relatives?; 3) Does variation in the COMT gene relate to neural functions implicated in higher-order cognition, and does this relationship differentiate genetic liability for schizophrenia from bipolar disorder?

Importantly, we contrasted event-related potentials elicited by stimuli during sensory control trials (i.e. “just look” and “press every”, see section 2.2; Sponheim et al., 2006) with trials on the DS-CPT to differentiate sensory responses to the images from responses evident during the vigilance demands of the DS-CPT. We hypothesized that vigilance effects would be most evident in early neural responses associated with perceptual processing of visual stimuli in individuals with liability for schizophrenia (Pokorny et al., 2019, Schallmo et al., 2013, Silverstein et al., 2015). Likewise, we compared ERPs elicited during nontarget and target trials of the DS-CPT to test whether impaired target detection differentiated genetic liability for schizophrenia from liability for bipolar disorder. Finally, we examined associations between ERPs and performance indices of the DS-CPT with medication dosage, and estimated IQ to investigate relationships with aspects of disease expression.

## 2 Method

### 2.1 Participants

Table 1 presents participant characteristics. Stable outpatients with schizophrenia or bipolar disorder, but no history of illicit drug dependence, were recruited from the clinics of the Minneapolis Veterans Affairs (VA) Medical Center, community support programs, and county mental health clinics. We identified first-degree biological relatives by interviewing patients and inviting them to participate via letter and telephone. Nonpsychiatric controls were recruited through postings at the Minneapolis VA Medical Center and surrounding Minneapolis community, and through newsletters for veterans and fraternal organizations. Exclusion criteria for control subjects included a personal or family history of psychosis or affective disorder (DSM-IV; American Psychiatric Association, 2000) and histories of substance dependence. Subjects were not excluded for history of alcohol dependence unless they had consumed alcohol in the last month. Both patient, and first-degree relative groups were not excluded for histories of substance dependence in order to adequately characterize families of schizophrenia probands. The relative number of subjects with a history of alcohol dependence is provided in Table 1**.** Notably, only patients with schizophrenia and controls differed in frequency (see Limitations). All participants gave informed consent, and study protocol and consent procedure were approved by both the Minneapolis VA Medical Center and University of Minnesota Institutional Review Boards. The COMT genotype of each participant was determined through a restriction fragment length polymorphism technique as described by Bergman-Jungeström and Wingren (2001) and detailed in Venables et al. (2009). A description can also be found in the Supplemental Materials.

**Table 1:** Characteristics of Participants.

Variable	Schizophrenia Patients	Bipolar Patients	Non-psychiatric Controls	Relatives of Schizophrenia Patients	Relatives of Bipolar Patients	Test Statistic	*p* Value
	n = 48	n = 26	n = 68	n = 55	n = 28		
	Mean (SD)	Mean (SD)	Mean (SD)	Mean (SD)	Mean (SD)		
Age (Years)	46.2 (8.6)	45.1 (9.8)	45.5 (11.9)	49.2 (9.3)	47.6 (13.9)	F(4,220) = 1.17	n.s.
Percent Female	17^a^, ^b^, ^c^	19^a^, ^b^, ^c^	48	58	50	χ^2^(4) = 26.11	<0.0005^1^
Year of Education	14.0 (2.6)^a^, ^b^	15.0 (1.5)	15.2 (2.0)	14.9 (2.3)	13.9 (3.0)^a^	F(4,220) = 3.14	0.01
Estimated IQ	97.2(11.2)^a^, ^b^, ^c^, ^d^	114.2 (14.9)	111.2 (10.7)	106.4 (13.7)^a^, ^d^	107.5 (12.9)^d^	F(4,220) = 11.68 < 0.0005	
BPRS Total Score	41.8 (11.1)^d^	34.9 (7.9)	NA	NA	NA	F(1,71) = 4.18	0.02
SPQ Total Score	NA	NA	9.7 (6.0)	14.0 (8.0)^a^	14.4 (13.9)^a^	F(2,140) = 4.71	0.01
CPZ Equivalents^2^	703 (5 5 5)	183 (1 4 0)	NA	NA	NA	NA	
History of Alcohol Dependence	23%^a^	12%	3%	11%	7%	χ^2^(4) = 12.21	0.018

*Note.* ANOVA = Analysis of Variance. SD = Standard Deviation. n.s. = not significant. IQ = Intelligence Quotient. Estimated IQ was derived from the formula of Brooker and Cyr (1986) using Vocabulary and Block Design subtests. BPRS = Brief Psychiatric Rating Scale (Ventura et al., 1993). NA = not applicable. SPQ = Schizotypal Personality Questionnaire (Raine, 1991). IQ data were missing from five control participants, eight relatives of schizophrenia patients, and five relatives of bipolar disorder patients. BPRS data were missing for one bipolar disorder proband. SPQ data were missing for two control participants, four relatives of schizophrenia patients, and two relatives of bipolar patients.

a Different from Control Group, *p* < .05.

b Different from Relatives of Schizophrenia Group, *p* < .05.

c Different from Relatives of Bipolar Group, *p* < .05.

d Different from Bipolar Group, *p* < .05.

1 Denotes significance level for Chi-Square test.

2 Reported for 45 schizophrenia patients and 6 bipolar disorder patients taking antipsychotic medications.

Trained doctoral-level clinical psychologists completed the Diagnostic Interview for Genetic Studies (DIGS; Nurnberger et al., 1994) and made symptom ratings using the Scale for the Assessment of Negative Symptoms (Andreasen, 1989), the Scale for the Assessment of Positive Symptoms (Andreasen, 1984), and the 24-item version of the Brief Psychiatric Rating Scale (Ventura et al., 1993) in order to inform clinical diagnoses. Relatives and control subjects completed the Structured Clinical Interview for DSM-IV Axis I Disorders (SCID-I; First et al., 1996) in addition to the schizotypal personality questionaire (SPQ, Raine, 1991). Lifetime diagnoses were determined through clinical consensus consistent with published guidelines (Leckman et al., 1982), which involved review of subjects’ clinical information from the study, requested medical history, and family informant material. All participants provided written consent after completing an informed consent procedure that included a demonstration of understanding of study procedures.

### 2.2 DS-CPT

**Fig. 1:**
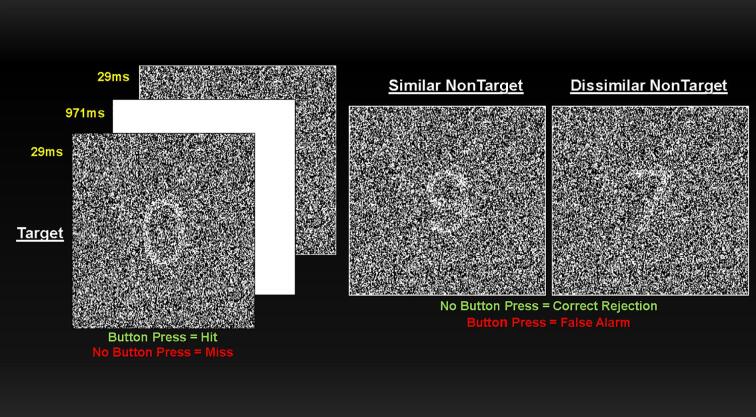
The Degraded Stimulus Continuous Performance Test. Each trial was composed of a single-digit numeral (4.3° × 3.4° visual angle in size) presented for 29 msec followed by a 971-msec white display. During “just look” trials, subjects passively viewed 80 trials of task stimuli. After a pause the experimenter instructed each subject to “press to every” stimulus for 80 trials. After completing control trials, subjects were instructed to press the button only when they thought they saw the numeral “0.” Twenty-five percent of the stimuli were targets (“0”), and 75% were nontargets (numerals “1” to “9”). After 160 practice trials, subjects were given a rest and then presented 480 continuous test trials over three blocks (160 trials per block) in a fixed-pseudorandom order. (*adapted from* K. H. Nuechterlein and Asarnow, 1999).

The version of the DS-CPT employed in the present study has been described previously (Continuous Performance Test Program for IBM-Compatible Microcomputers, Version 7.10 for the Degraded Stimulus CPT, Nuechterlein and Asarnow, 1999, Sponheim et al., 2006; Fig. 1). Briefly, task stimuli and background were degraded; 40% of white numeral pixels were switched to black, and 40% of black background pixels switched to white. Sensory control trials were administered before DS-CPT instructions were provided and consisted of “just look” (participants instructed to look passively at the screen) and “press every” (participants instructed to respond to each stimulus) at 80 trials each. Following a practice block, subjects then received DS-CPT instructions and completed three experimental blocks wherein 25% of stimuli were targets (“0”) and the remainder were nontargets (numerals “1” to “9”). Trials designated as “similar” more closely resembled targets (“6”, “8” “9”), while “dissimilar” consisted of the remaining numerals. For practice and experimental blocks, participants were told to respond only when they saw targets. The standard signal detection index dˊ was computed for each subject.

### 2.3 Electrophysiological data collection and analyses

EEG collection procedures for participants has been detailed previously (Sponheim et al., 2006). Electroencephalogram signals were digitized at a rate of 500 Hz with a 0.05-Hz low-frequency, 100-Hz high-frequency, and a 60-Hz notch filter. Recordings were divided into epochs extending from 100 msec before stimulus to 1000 msec post-stimulus. Vertical electro-oculograms were used to remove ocular artifact (Semlitsch et al., 1986) and data baseline corrected to 100 msec prior to the onset of the stimulus, and filtered with a high-frequency cutoff of 30 Hz (48 dB/octave roll-off) and a low-frequency cutoff of 0.1 Hz (48 dB/octave roll-off). Epochs with signals exceeding ± 100 μV or horizontal electrooculogram were automatically rejected. All epochs were visually inspected, and excluded if artifacts<100 μV were identified. Only trials containing correct responses were averaged for control (just look, button press), target, and nontarget trials. For those components for which individuals exhibited a clear peak, we measured peak amplitude (N1 [140–200 msec], P3b [360 – 700 msec]). Difference waveforms were used for the peak N2 [320–400 msec] component, as previous reports have demonstrated reductions in patients with schizophrenia involving cognitive processes related to impaired target detection (Ford et al., 2010, Kasai et al., 1999, Salisbury et al., 1994, Swainson et al., 2003). N2 difference waveforms consisted of peak nontarget amplitude subtracted from peak target amplitude. We included electrodes O1 and O2 for the N1 component, and electrodes Cz and Fz for the N2 difference waveform, as the components were most evident at these electrode sites across groups. For the P3b component, we used electrodes P7 and P8 to keep analyses consistent with the key finding from our previous report (Sponheim et al., 2006), where the component was most evident at electrode sites P7 and P8 for relative groups.

### 2.4 Statistical analyses

To examine how neural components associated with target detection during vigilance and sensory control trials are associated with genetic liability for schizophrenia and bipolar disorder, we conducted two sets of multivariate analyses of variance (MANOVAs) with two between-subjects factors (group and gender) for each ERP component of interest (i.e. N1, N2, P3b). In one analysis set, the group factor consisted of healthy controls (HC), patients with schizophrenia (SZ) and first-degree relatives of patients with schizophrenia (SZRel), while the second set consisted of HC, bipolar probands (BP) and first-degree relatives of bipolar patients (BPRel). For analyses relating to N1, three within-subject factors were used (target: target vs. nontarget, hemisphere: left vs. right, task: vigilance vs. sensory control trials) because the component was evident across all task conditions. Only one within-subjects factor (region: electrode Cz vs. electrode Fz) was used for analyses examining N2 difference waveforms because the component was only evident during vigilance trials. Finally, because P3b was only evident to target objects, the within-subjects factors of hemisphere and task (vigilance vs. “press only” trials) were used to examine how the vigilance and target identification demands of the DS-CPT affected the component beyond the motor response demands of both trial types. In order to account for the shared genetic contribution present amongst individual families biasing our results, we performed mixed effects models with family membership included as a random effect (see Supplementary Table S1). Given the large number of within-subject variables, we corrected for multiple comparisons involving these variables using Šídák corrections, which are less conservative than Bonferroni corrections. In order to test *a priori* hypotheses involving perceptual functions in schizophrenia, and to avoid unduly penalizing power in our tests for group differences, we corrected multiple comparisons by controlling for the false discovery rate (FDR; Benjamini and Hochberg, 1995).

We computed correlations between behavioral indices on the DS-CPT (i.e. dˊ, rate of false alarms, and SimDiff) and N1 amplitude at electrode O2 (where peaks were greatest) for each group to examine associations between early visual processes and performance related to perceptual processing of stimuli. In addition, we correlated demographic variables of IQ, Chlorpromazine equivalence, and behavioral indices on the DS-CPT (i.e. dˊ, SimDiff) with both N2 difference amplitude at Cz, and P3b amplitude at P7 within each group. To address possible inflation of Type I error for the computed correlations, we again employed the Benjamini-Hochberg procedure to correct for the False Discovery Rate.

## 3 Results

### 3.1 Task performance

Behavioral results as well as contrasts involving all groups and participants are displayed in Table 2. A closer examination of the distribution of dˊ scores revealed 3 exceptionally high-performing outliers among SZ, and one among SZRel according to procedures outlined in Schwertman et al. (2004). There were no other behavioral outliers in any other group. Planned comparisons between HC and SZ after removing said outliers revealed SZ performed worse than HC on block 2 (FDR corrected *p* = .024) and overall (FDR corrected *p* = .044). Removal of the high-performing relative did not significantly change the mean dˊ value for SZRel (FDR corrected *p* = .614). We directly compared participant characteristics between SZ in the present sample, and SZ from the sample in our previous report—a subset of the present sample was presented in our 2006 paper—to account for possible differences in demographics perhaps related to better task performance (see Supplementary Table 2).

**Table 2:** Degraded Stimulus Continuous Performance Task (DS-CPT) Performance.

Task Value^1^		Schizophrenia Patients	Bipolar Patients	Non–psychiatric Controls	Relatives of Schizophrenia Patients	Relatives of Bipolar Patients	ANOVA Test Value	Effect Size-η^2^	*p Value*	
		n = 48	n = 26	n = 68	n = 55	n = 28				
		Mean (SD)	Mean (SD)	Mean (SD)	Mean (SD)	Mean (SD)				
Target Detection: d′										
	Block 1	2.67 (0.89)	2.67 (1.08)	3.02 (1.06)	2.85 (0.89)	2.23 (1.20)^a^, ^b^	F(4,220) = 3.25	0.046	0.01	
	Block 2	2.38 (0.96)	2.40 (1.14)	2.75 (1.10)	2.59 (0.86)	1.97 (1.00)^a^, ^b^	F(4,220) = 3.31	0.047	0.01	
	Block 3	2.27 (0.96)	2.21 (0.93)	2.49 (1.08)	2.56 (0.88)	1.96 (0.87)^a^, ^b^	F(4,220) = 2.37	0.041	0.05	
	Total	2.42 (0.93)	2.37 (0.98)	2.73 (1.10)	2.64 (0.89)	1.99 (0.89)^a^, ^b^	F(4,220) = 3.37	0.05	0.01	
Similar-Dissimilar False Alarms										
		12.89 (9.6)	11.69(10.5)	9.70(10.9)	16.03(10.2)^a^	10.3(12.4)	F(4,220) = 2.81	049	03	
Reaction Time to Targets: (msec)										
	Total	529 (88) a, b	531 (68) a, b	481 (61)	493 (55)	548 (95) a, b	F(4,220) = 6.75	0.109	<0.001	

*Note.* ANOVA = Analysis of Variance. SD = Standard Deviation. n.s. = not significant.

1 Denotes significance level of One-way ANOVA

a Different from Control Group mean, Tukey’s HSD *p* ≤ 0.05

b Different from Relatives of Schizophrenia Group mean, Tukey’s HSD *p* ≤ 0.05

In order to account for the unequal distribution of male and female subjects in proband groups and to test for whether performance deficits were specific to liability for a particular disorder, or indicative of liability for severe mental illness in general, two 3 × 2 Mixed Model ANOVAs were fitted with between-subjects factors of group and gender, and a within-subjects factor of experimental block (first, second, and third). The first ANOVA tested differences between HC, SZ and SZRel. There was a main effect of block (F_(_*_2, 161)_* = 56.09, *p* < .001, η^2^ = 0.258), with average performance decreasing on later blocks, with no effect of group (F_(_*_2, 161)_* = 2.05, *p* = .132, η^2^ = 0.025), gender (F_(_*_1, 165)_* = 0.190, *p* = .542, η^2^ = 0.001) and no observed interaction between group and gender (F_(_*_2, 165)_* = 0.717, *p* = .74, η^2^ = 0.004). The second ANOVA examined differences between HC, BP and BPRel. There was a main effect of block (F_(_*_2, 116)_* = 17.21, *p* < .001, η^2^ = 0.129), group (F_(_*_2, 116)_* = 5.41, *p* < .01, η^2^ = 0.086), with no effect of gender (F_(_*_1, 116)_* = 2.39, *p* = .125, η^2^ = 0.020) and an interaction between group and gender (F_(_*_2, 116)_* = 3.12, *p* = .048, η^2^ = 0.051), as well as an interaction between block, group and gender (F_(_*_2, 116)_* = 4.00, *p* = .014, η^2^ = 0.052). Simple main effects revealed that performance deficits were specific to male BP (M = 2.21, SE = 0.212; Šídák corrected *p =* .05) and male BPRel (M = 2.00, SE = 0.259; Šídák corrected *p =* .018) compared to male HC (M = 2.86, SE = 0.164), with the greatest differences occurring during blocks 1 and 2 of the task. Across all subjects, total dˊ was associated with IQ (*r*(2 0 7) = 0.31, FDR corrected *p <* .01), and this association was also seen in BP (*r*(26) = 0.60, FDR corrected *p* = .02) as well as SZRel (*r*(47) = 0.55, FDR corrected *p* < .01). DS-CPT performance indices failed to be associated with symptom measures in patients and schizotypy scales (SPQ) in relatives.

To examine the hypothesis that perceptual processes related to contour detection affect performance in individuals with genetic liability for schizophrenia, we carried out exploratory analyses of the rate of incorrect target identifications for nontargets that shared curved contours with the target ‘0′ (numerals ‘6′, ‘8′ and ‘9′). Two 3 × 2 ANOVAs with between subjects factors of group and gender were employed to examine the rate of false alarms to similar nontargets while subtracting errors for dissimilar nontargets to control for overall false alarm rate (i.e., SimDiff Errors; Table 2). The first ANOVA examining healthy controls, patients with schizophrenia and their first-degree relatives yielded a main effect of group (F_(_*_2, 167)_* = 3.19, *p* = .01, η^2^ = 0.054). Follow-up post-hoc tests revealed SZRel had greater SimDiff errors than HC (FDR corrected *p <* .01*)* while the errors for SZ failed to be significantly higher (FDR corrected *p* = .362). A separate 3 × 2 ANOVA examining healthy controls, BP and BPRel yielded a significant effect of gender (F_(1, 116)_ = 3.19, p = .047, η^2^ = 0.034) and an interaction between group and gender (F_(2, 116)_ = 3.36, *p* = .038, η^2^ = 0.055). Follow-up simple main effects tests revealed that female BPRel (M = 16.07, SE = 2.60) had substantially more SimDiff errors than male BPRel (M = 4.5, SE = 2.60; Šídák corrected *p* < .01).

### 3.2 ERP results

#### 3.2.1 Neural anomalies and genetic liability for schizophrenia

Early posterior potential: N1

**Fig. 2:**
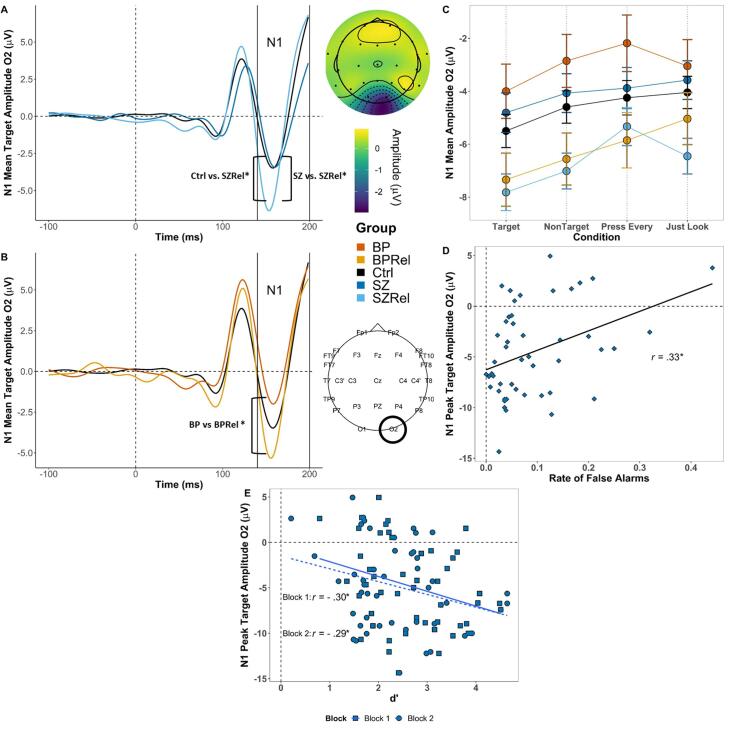
Grand averaged N1 to targets at O2. Topographies depict peak N1 amplitude across the N1 window (140–200 msec) in control subjects. (A) SZRel had greater peak amplitude compared to HC (FDR corrected *p* = .035) and SZ (FDR corrected *p* = .015. (B) BPRel had greater peak amplitude compared to BP (FDR corrected *p* = .014) but not HC (FDR corrected *p* = .119). (C) N1 amplitudes across groups and conditions at O2 (error bars ± SEM). (D and E) N1 amplitude to targets at O2 was positively associated with the number of false alarms (*r(48) = 0.*33, FDR corrected *p* = .04; D) and dˊ in block 1 (*r*(48) *=* -0.30, FDR corrected *p* = .04) and block 2 (*r*(48) *= -*0.29*,* FDR corrected *p* = .05; E) in SZ. * indicates p < .05 | ** indicates *p* < .01.

A MANOVA examining the N1 component in HC, SZ and SZRel at O1 and O2 revealed a main effect of task (*F*_(_*_1,161)_* = 19.60, *p* < .001, Wilk’s Λ = 0.891, partial η^2^ = 0.109), where N1 was greater during vigilance (M = -5.42, SE = 0.42) than sensory control trials (M = -4.43, SE = 0.394; Šídák corrected *p* < .001). There was also an interaction between task, group and gender (*F*_(_*_2,161)_* = 4.25, *p* = .016, Wilk’s Λ = 0.95, partial η^2^ = 0.05). Follow-up simple main-effects analyses revealed that N1 amplitudes were greater to target (Šídák corrected *p* < .001) and nontarget trials (Šídák corrected *p* = .032) during vigilance than for sensory control trials, particularly at electrode site O2 (Šídák corrected *p* < .001). There were non-significant effects of gender (F_(1,161)_ = 3.38, p = .068, η^2^ = 0.024), a main effect of group (F_(2,161)_ = 2.54, *p* = .032, η^2^ = 0.031), and no interaction between group and gender (F_(2,161)_ = 0.762, *p* = .468, η^2^ = 0.009). Post-hoc comparisons revealed that SZRel had greater N1 amplitudes than both HC (FDR corrected *p* = .03) and SZ (FDR corrected *p* = .02.) with this difference being greatest at site O2 for targets during vigilance (Fig. 2A). Additional follow-up simple main effects analyses revealed that female SZRel (M = -7.81, SE = 0.833) differed from female HC during sensory control trials (M = -5.84, SE = 0.83; Šídák corrected *p =* .02). The effect of N1 across conditions is presented in Fig. 2C (an alternate version of the figure with jittered points is presented as Supplementary Fig. 1). A main effect of hemisphere was also observed (*F*_(_*_1,161)_* = 6.77, *p* = .01, Wilk’s Λ = 0.987, partial η^2^ = 0.04) such that amplitudes were greater at O2 (i.e. right hemisphere; M = -5.17, SE = 0.42) than at O1 (M = -4.68, SE = 0.39; Šídák corrected *p* = .01). There were interactions between task and target (*F*_(_*_1,161)_* = 5.48, *p* = .02, Wilk’s Λ = 0.967, partial η^2^ = 0.03) and between task and hemisphere (*F*_(_*_1,161)_* = 4.02, *p* = .047, Wilk’s Λ = 0.976, partial η^2^ = 0.024).

In SZ, N1 amplitude at electrode site O2 was positively associated with the false alarm rate (*r*(48) = 0.33, FDR corrected *p* = .04; Fig. 2D), suggesting that *reduced* augmentation of early posterior brain responses to targets contributed to a tendency to identify nontarget stimuli as targets. N1 amplitudes were additionally negatively correlated with dˊ during block 1 (*r*(48) *=* -0.30, FDR corrected *p* = .04) and block 2 (*r*(48) *= -*0.29*,* FDR corrected *p* = .05) in SZ also suggesting that robust early posterior responses to stimuli yielded better differentiation of target and nontarget stimuli (Fig. 2E).

Middle latency potential: N2

**Fig. 3:**
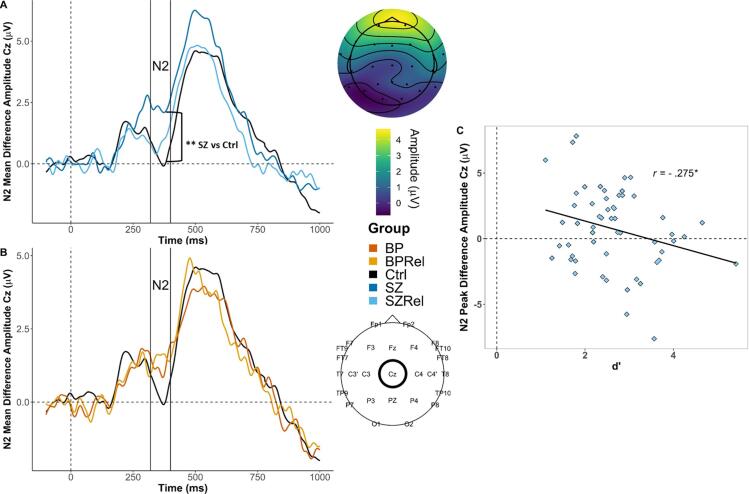
Grand averaged N2 difference waveforms (targets – nontargets) at Cz. Topography depicts mean N2 difference amplitudes across the N2 time window (320–400 msec) in control subjects. (A) SZ had reduced peak amplitudes compared to HC (FDR corrected *p* < .01). (B) There were no observed group differences in peak N2 difference waveforms between BP, BPRel and HC. (C) N2 amplitudes were positively associated with average dˊ in SZRel (*r(54) =* -0.275, FDR corrected *p* = .05). * indicates p < .05 | ** indicates *p* < .01.

A MANOVA examining group differences between HC, SZ and SZRel revealed a main effect of group (F_(2,161)_ = 5.30, p < .01, η^2^ = 0.061) with follow-up post-hocs revealing that SZ had reduced amplitudes compared to HC (FDR corrected *p* < .01), with this difference being greatest at site Cz to targets during vigilance (Fig. 3A). There was a non-significant effect of gender (F_(1,161)_ = 2.85, p < .093, η^2^ = 0.017), and no interaction between gender and group (F_(2,161)_ = 0.251, p = .778, η^2^ = 0.003). There were also interactions between electrode site and group (*F*_(_*_2,162)_* = 5.33, *p* < .01 , Wilk’s Λ = 0.938, partial η^2^ = 0.062) and electrode site and gender (*F*_(_*_1,161)_* = 4.19, *p* = .042 , Wilk’s Λ = 0.938, partial η^2^ = 0.025). An effect of electrode (*F*_(_*_1,1621)_* = 61.42, *p* < .001 , Wilk’s Λ = 0.725, partial η^2^ = 0.275) reflected that N2 difference waveforms were greater at Cz (M = -8.14, SE = 0.308) than Fz (M = 0.595, SE = 0.246; Šídák corrected *p <* .001). Follow-up analyses for each electrode site revealed that SZ had reduced N2 difference waveforms at Cz (M = 1.307, SE = 0.534) compared to HC (M = -0.240, SE = 0.336; Šídák corrected *p* = .045) and reduced N2 difference waveforms at Fz (M = 0.831, SE = 0.67) compared to both HC (M = -2.00, SE = 0.422; Šídák corrected *p* < .01) and SZRel (M = -1.28, SE = 0.48; Šídák corrected *p* = .035). Further simple main effects analyses showed that females had greater difference waveforms at Cz (M = -0.185, SE = 0.497) than males (M = 0.857, SE = 0.396; Šídák corrected *p* = .045).

In SZRel, there was a significant association between total dˊ (across all three blocks) and N2 difference waveforms at site Cz (*r(54) =* -0.275, FDR corrected *p* = .05), such that larger N2 responses to target stimuli were associated with better differentiation between target and nontarget stimuli on the DS-CPT (Fig. 3C).

Late posterior potential: P3b

**Fig. 4:**
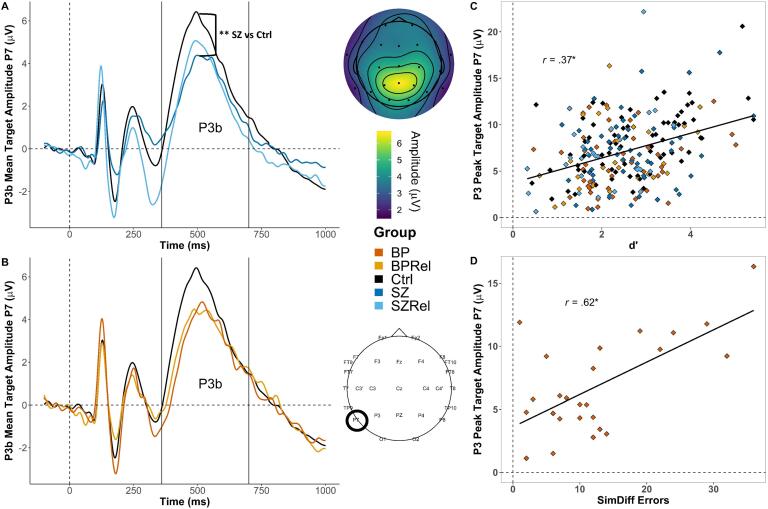
Grand averaged P3b amplitude to targets at P7. Topography depicts mean P3b amplitudes across the P3b time window (320–400 ms) in control subjects. (A) SZ had reduced peak amplitudes compared to HC (FDR corrected *p* < .01). (B) There were no observed group differences in peak P3b amplitudes between BP, BPRel and HC at P7 alone.(C) P3b amplitudes were positively associated with average dˊ across all five groups (*r*(2 2 5) = 0.37, FDR corrected *p <* .01). (D) P3b amplitudes were positively associated with the number of SimDiff errors in BP (*r*(25) = 0.619, FDR corrected *p* < .01). * indicates *p* < .05 | ** indicates *p* < .01.

A MANOVA examining the P3b component to targets at electrode sites P7 and P8 in HC, SZ and SZRel revealed a significant effect of task (*F*_(_*_1,162)_* = 167.02, *p* < .001, Wilk’s Λ = 0.492, partial η^2^ = 0.508) where amplitudes were greater during vigilance (M = 7.23, SE = 0.29) relative to “press every” control trials (M = 3.12, SE = 0.178; Šídák corrected *p* < .001). There were also main effects of group (*F_(2,162)_* = 3.14, *p* = .016, partial η^2^ = 0.05), gender (*F_(1,162)_* = 6.031, *p* = .015, partial η^2^ = 0.036), and an interaction between gender and group (*F_(2,162)_* = 3.161, *p* = .045, partial η^2^ = 0.038). Post-hoc comparisons revealed that SZ and SZRel had smaller P3bs than HC (FDR corrected *p <* .01*;* FDR corrected *p =* .023 respectively*)*, particularly at electrode site P7 for target stimuli during vigilance (Fig. 4A). There was also an interaction between hemisphere and group (*F*_(_*_2,162)_* = 3.14, *p* = .046, Wilk’s Λ = 0.963, partial η^2^ = 0.204), with SZ (M = 4.43, SE = 0.413) having reduced P3b components compared to HC in the left hemisphere (i.e. P7; M = 5.92, SE = 0.262; Šídák corrected *p* < .01) and SZRel (M = 4.94, SE = 0.295). SZ also displayed reduced P3b compared to HC at P7 (Šídák corrected *p =* .042). Follow-up simple main effects revealed that gender differences were only apparent in HC (*F_(1,162)_* = 11.454, *p* < .041, partial η^2^ = 0.066), with females having greater P3b compared to males (Šídák corrected *p* < .01).

The only association specific to SZ was that larger P3b amplitude at electrode site P7 was associated with higher daily dosages for antipsychotic medication (chlorpromazine equivalence; *r*(45) *=* 0.412, FDR corrected *p <* .01).

#### 3.2.2 Neural anomalies and genetic liability for bipolar disorder

Early posterior potential: N1

A MANOVA examining N1 amplitudes at O1 and O2 in HC, BR and BPRel revealed a main effect of task (*F*_(_*_1,111)_* = 10.67, *p* < .01, Wilk’s Λ = 0.912, partial η^2^ = 0.088), with N1 responses being greater during vigilance (M = -4.84, SE = 0.50) than sensory control trials (M = -3.77, SE = 0.329; Šídák corrected *p* < .01). There was also an effect of group (*F_(2,111)_* = 3.31, *p* = .04, partial η^2^ = 0.056), with follow-up post hoc tests revealing that BPRel had greater N1 amplitudes than BP (FDR corrected , *p* = .033). Group differences were maximal at electrode O2 to targets during vigilance (Fig. 2B). Correlations were computed between N1 amplitude at site O2 with no correlations surviving FDR correction.

Middle latency potential: N2

A MANOVA examining N2 difference waveforms at electrode sites Cz and Fz revealed an effect of region (*F*_(_*_1,115)_* = 49.55, *p* < .001, Wilk’s Λ = 0.70, partial η^2^ = 0.301), with N2 amplitudes being greater at Cz (M = -1.18, SE = 0.33) than Fz (M = 0.405, SE = 0.326; Šídák corrected *p* < .001). There was no effect of group (*F_(2,115)_* = 1.96, *p* = .145, partial η^2^ = 0.033), gender (*F_(1,115)_* = 2.73, *p* = .101, partial η^2^ = 0.023) and no interaction between group and gender (*F_(1,115)_* = 0.524, *p* = .593, partial η^2^ = 0.009). Planned comparisons revealed no group differences after FDR correction **(**Fig. 3B). No correlations in patients with bipolar disorder or their first-degree relatives survived FDR correction.

Late posterior potential: P3b

A MANOVA examining P3b amplitudes to targets at electrode sites P7 and P8 compared HC, BP and BPRel. There was a main effect of task (*F*_(_*_1,112)_* = 70.65, *p* < .001, Wilk’s Λ = 0.613, partial η^2^ = 0.387), with P3b amplitudes being greater during vigilance (M = 7.08, SE = 0.35) than “press every” trials (M = 3.57, SE = 2.54; Šídák corrected *p* < .001). There were also interactions between hemisphere and gender (*F*_(_*_1,112)_* = 5.54, *p* = .02, Wilk’s Λ = 0.953, partial η^2^ = 0.047) and hemisphere and task (*F*_(_*_1,112)_* = 11.92, *p* < .01, Wilk’s Λ = 0.904, partial η^2^ = 0.096). There were also main effects of group (*F_(2,112)_* = 4.137, *p* = .018, partial η^2^ = 0.060), gender (*F_(1,112)_* = 6.28, *p* = .014, partial η^2^ = 0.053) and an interaction between gender and group (*F_(2,112)_* = 3.31, *p* = .04, partial η^2^ = 0.056). Follow-up analyses revealed that female BPRel had reduced P3b amplitudes compared to both HC (Šídák corrected *p* < .01) and BP (Šídák corrected *p* = .048). Post-hoc comparisons revealed HC had greater P3b amplitudes compared to BPRel (FDR corrected *p* = .015), and a non-significant modulation relative to BP (FDR corrected *p* = .071), with the greatest differences observed at electrode site P7 during vigilance (Fig. 4B). There was also an interaction between task, side and group (*F*_(_*_2,112)_* = 3.55, *p* = .032; Wilk’s Λ = 0.94, partial η^2^ = 0.06) with follow-up simple main effects analyses revealing BPRel had smaller P3b amplitudes at both parietal electrodes (site P7 - BPRel: M = 6.27, SE = 0.622; HC: M = 8.15 SE = 0.387; Šídák corrected *p* = .034; site P8 – BPRel: M = 5.64, SE = 0.64; HC: M = 7.94, SE = 0.399; Šídák corrected *p* < .01). Additional follow-up analyses revealed females had greater P3b amplitudes in the left hemisphere (i.e. P7; M = 6.09, SE = 0.38) compared to males (M = 4.58, SE = 0.267; Šídák corrected *p* < .01), and that during vigilance subjects had greater P3b amplitudes at P7 (M = 7.32, SE = 0.36) than P8 (M = 6.83, SE = 0.37; Šídák corrected *p* = .018). In contrast, subjects had greater P3b amplitudes during “press every” trials at P8 (M = 3.35, SE = 0.275) than P7 (M = 3.35, SE = 0.275; Šídák corrected *p < .*045).

Across all groups (i.e. HC, SZ, SZRel, BP and BPRel) larger P3b amplitudes at electrode P7 were related to better target stimulus detection on the DS-CPT (dˊ: *r*(2 2 5) = 0.37, FDR corrected *p <* .01; Fig. 4C) and estimated IQ (*r*(2 0 7) *=* 0.25, FDR corrected *p* < .01). The association between P3b amplitude and performance was also evident in HC (dˊ: *r*(68) *=* 0.514, FDR corrected *p* < .01), as was fewer misidentifications of nontarget stimuli as targets (*r*(68) *=* -0.368, FDR corrected *p* < .01). Interestingly, in BP P3b amplitudes were associated with a tendency to confuse similar nontarget stimuli with targets (SimDiff errors: *r*(25) = 0.619, FDR corrected *p* < .01; Fig. 4**D)** possibly suggesting that the late ERP for this group reflected the perception that the nontarget stimulus was a target.

### 3.3 COMT genotype and ERP results

Given that COMT polymorphisms are generally implicated in prefrontal cortical networks involved in cognitive control and working memory processes (Cools and D’Esposito, 2011) we focused on the associations between COMT with ERP components previously associated with cognitive processes (N2 and P3b). To restrict analyses, we only examined the associations between COMT and N2 in relation to schizophrenia given the lack of group differences in the bipolar disorder-related samples; these analyses failed to yield any effects involving genotype (see Supplementary Materials). Additionally, there were no female SZ that were met homozygotes and only a single female BP that was a val/met heterozygote which precluded the inclusion of gender as a factor in statistical analyses of COMT variation (i.e., 3 X 2 Mixed Model MANOVAS with the factors of group and genotype were used).

A MANOVA examining P3b effects at P7 and P8 among HC, SZ and SZRel included a genotype distribution of val homozygotes (*N =* 34), val/met heterozygotes (*N =* 61) and met homozygotes (*N =* 28). There was a main effect of genotype (*F*_(_*_1,114)_* = 3.37, *p* = .035, partial η^2^ = 0.056), but no effect of diagnostic group (*F*_(_*_2,114)_* = 2.05, *p* = .134, partial η2 = 0.035), and no interaction between diagnostic group and genotype (*F*_(_*_2,114)_* = 1.12, *p* = .352 , partial η^2^ = 0.038). The analysis also revealed an effect of task (*F*_(_*_1,114)_* = 133.88, *p* < .001, Wilk’s Λ = 0.46, partial η^2^ = 0.54), with P3b amplitudes being greater during vigilance (M = 6.81, SE = 0.33) than “press every” trials (M = 3.13, SE = 0.17; Šídák corrected *p* < .001). There was also an interaction between task and genotype (*F*_(_*_2,114)_* = 4.11, *p* = .019, Wilk’s Λ = 0.46, partial η^2^ = 0.54) with val homozygotes (M = 5.42, SE = 0.60) having reduced P3b amplitudes during vigilance compared to val/met heterozygotes (M = 7.01, SE = 0.43; Šídák corrected *p* = .034) and met homozygotes (M = 8.02, SE = 0.64; Šídák corrected *p* < .01)

A MANOVA examining P3b effects at P7 and P8 among HC, BP and BPRel included a genotype distribution of val homozygotes (*N =* 17), val/met heterozygotes (*N =* 40) and met homozygotes (*N =* 20). There were no main effects of group (*F*_(_*_2,68)_* = 1.012, *p* = .36; partial η^2^ = 0.03) nor genotype group (*F*_(_*_2,68)_* = 1.01, *p* = .37; partial η^2^ = 0.03), and a non-significant interaction between diagnostic group and genotype (*F*_(_*_2,68)_* = 2.48, *p* = .051; partial η^2^ = 0.128). There was an effect of task condition (*F*_(_*_1,68)_* = 90.24, *p* < .001; Wilk’s Λ = 0.43, partial η^2^ = 0.57) with P3b amplitudes being greater during vigilance (M = 6.80, SE = 0.35) than “press every” trials (M = 3.13, SE = 0.25; Šídák corrected *p* < .001) and an interaction between task and genotype (*F*_(_*_1,68)_* = 5.47, *p* < .01;Wilk’s Λ = 0.861, partial η^2^ = 0.14). Follow-up simple main effects tests revealed that met homozygotes (M = 8.04, SE = 0.64) had greater P3b amplitudes than val/met heterozygotes (M = 6.01, SE = 0.45; Šídák corrected *p =*.03). There was also an interaction between hemisphere and task (*F*_(_*_1,68)_* = 6.97, *p* < .001; Wilk’s Λ = 0.91, partial η^2^ = 0.093), but follow-up simple main effects analyses did not survive correction for multiple comparisons.

We examined associations between neural functions (i.e. N2 and P3b), behavioral (dˊ) and demographic indices (i.e. IQ and Chlorpromazine equivalence) for each genotype within samples related to schizophrenia (i.e. patients with schizophrenia and first-degree relatives)and bipolar disorder (i.e. patients with bipolar disorder and first-degree relatives) in order to evaluate the relationship between COMT genotype and P3b amplitude. For val homozygotes with liability for schizophrenia, total dˊ across blocks was positively associated with P3b amplitude to targets at site P7 (*r(*28) *=* 0.456, FDR corrected *p* = .04). In individuals with liability for bipolar disorder, correlations between DS-CPT performance and neural functions did not survive FDR correction. To examine the effects of medication on P3b, we examined these associations across genotype groups within the two patient groups. Chlorpromazine equivalence was positively associated with P3b in schizophrenia probands that were val homozygotes (*r*(15) = 0.622, FDR corrected *p* = .04). There were no associations between P3b and medication in bipolar probands.

## 4 Discussion

In this study, we examined behavioral performance and neurophysiological responses to the DS-CPT in healthy controls, patients with schizophrenia, patients with bipolar disorder, and first-degree biological relatives of patients with both disorders to determine whether previously reported abnormalities in brain responses were associated with genetic liability for bipolar disorder as well as schizophrenia. First-degree relatives of both patient types exhibited augmented N1 components compared to respective patient groups suggesting possible early compensatory visual functions during vigilance for visual targets that are perceptually difficult to discern. Diminished N2 difference waveforms were specific to schizophrenia patients and may reflect disorder-specific deficits in high-level object recognition involving perception of complex stimuli (S. J. Luck et al., 2009) or sensitivity to the degree of perceptual deviation of targets from nontargets (Folstein and Petten, 2008). Both patients with schizophrenia and their relatives exhibited reduced P3b components compared to controls and therefore the component may tap aspects of genetic liability for schizophrenia (i.e., be an endophenotype). Neurophysiological responses of context processing (P3b) were associated with COMT gene variation. COMT val homozygotes had the smallest P3b amplitudes when examining liability for schizophrenia, whereas val/met heterozygotes had the smallest P3b amplitudes with respect to liability for bipolar disorder, suggesting COMT variation may differentially influence neural functions indicative of higher-order cognition across the two severe mental disorders.

Relative groups of both types exhibited augmented N1 components across vigilance and sensory control conditions in contrast to comparable patient groups, though only relatives of patients with schizophrenia had larger N1 amplitudes than healthy controls. In relatives of patients with schizophrenia, augmented N1 appears to be a compensatory component given 1) relatives demonstrated intact performance on the DS-CPT; and 2) augmented N1 was associated with better performance in patients with schizophrenia suggesting a beneficial role of early visual cortical responses in discriminating degraded visual stimuli. This finding is consistent with a recent report in which augmented ERPs at 200 msec (comparable to N1) to target verniers in a backward masking task reflected neural compensation in relatives of patients with schizophrenia (da Cruz et al., 2020). In contrast, we do not interpret N1 as compensatory in the context of relatives of bipolar patients given the lack of observed associations between N1 and performance in both patients with bipolar disorder or first-degree relatives, and that relatives’ N1 potentials did not differ from healthy controls.

Results also suggest that diminished N2 difference waveforms are specific to patients with schizophrenia. Previous work has demonstrated that reductions in N2 potentials reflect a broad attentional deficit in schizophrenia (Salisbury et al., 1994), with deficits in N2 difference waveforms related to a dysfunction in classification of stimuli as targets during stimulus identification (Wood et al., 2006). Consistent with a target identification function, larger N2 difference waveforms in relatives of patients with schizophrenia were associated with greater dˊ, suggesting that neural responses differentiating targets and nontarget stimuli facilitate DS-CPT behavioral performance. Importantly, the present findings indicate that deficits in fronto-central N2 responses reflect deficits in neural functions related to visual object recognition that are specific to schizophrenia pathology (Doniger et al., 2002). Alternatively, the deficit could be in the N2pb component at parieto-occipital sites, reflecting a deficit in visual classification that is specific to schizophrenia (S. J. Luck and Hillyard, 1994).

The present study replicated previous findings of impaired P3b in both patients with schizophrenia and their relatives suggesting deficits in neural functions implicated in target detection may constitute an endophenotype of schizophrenia (Groom et al., 2008, Knott et al., 1999, Roxborough et al., 1993, Sponheim et al., 2006). In contrast, deficits in P3b were specific to relatives of patients with bipolar disorder. Critically, the observed interaction between gender and diagnostic group in models examining specificity for schizophrenia appeared to be driven by sex differences in healthy controls, consistent with the idea that healthy female controls have larger P3b components compared with males (Conroy and Polich, 2007, Steffensen et al., 2008). This same interaction in models examining the specificity of bipolar disorder revealed that female relatives had reduced P3b components compared to both healthy controls and bipolar probands. Results from the present work suggest abnormalities in fronto-posterior parietal networks constitute an endophenotype for schizophrenia, and may be indicative of neural expression of genetic liability for bipolar disorder in female relatives.

Variation in the COMT genotype was not strongly related to differences in neural responses between the clinical disorders of schizophrenia and bipolar disorder. However, when examining genetic liability for schizophrenia, val homozygotes had the most pronounced deficits in P3b amplitudes, consistent with findings that increased dopaminergic catabolism in prefrontal neurons impairs higher order cognition in schizophrenia (Bilder et al., 2002, Roffman et al., 2008, Shifman et al., 2002). In contrast, when examining genetic liability for bipolar disorder, val/met heterozygotes had the smallest P3b amplitudes relative to met/met homozygotes. These results align somewhat with our previous report in which val and met homozygotes had differential associations with clinical symptomatology in schizophrenia and bipolar disorder respectively (Goghari and Sponheim, 2008). Given that met homozygotes generally outperform the other genotypes in tasks tapping higher-order processing (Bruder et al., 2005, Tsai et al., 2003), our present findings suggest higher order cognition may be differentially impacted by COMT polymorphisms across liability for schizophrenia and bipolar disorder.

Contrary to expectations, we did not observe diminished perceptual sensitivity in patients with schizophrenia. In the new sample (i.e. the sample from the present study as opposed to our 2006 study) , dˊ was more similar to what would be expected in healthy controls (Nuechterlein et al., 2015), and was significantly greater than we observed in a subset of the present sample from our laboratory. Patients with schizophrenia in the present sample had a larger daily dose of chlorpromazine equivalence. Given the positive association between P3b amplitudes to targets and chlorpromazine equivalence in the present sample, higher doses of medication may have improved neural functions associated with target detection (Sponheim et al., 2006), indicating that medication effects in the present sample may mark effective treatment rather than disease severity (Nuechterlein et al., 2015). Earle-Boyer et al. demonstrated that unmedicated patients made more errors than medicated patients when performing various CPTs regardless of stimulus modality (Earle-Boyer et al., 1991), and a *meta*-analysis has provided evidence that antipsychotic medication mitigated P300 deficits in patients with schizophrenia (Bramon, 2004). Critically, when three outliers for performance were considered, patients with schizophrenia performed more poorly than healthy controls.

Our findings of a male specific decrement in dˊ in bipolar probands and their relatives is consistent with previous reports documenting reduced attention and visual discrimination in male bipolar patients (Barrett et al., 2008, Gogos et al., 2010), and that vigilance deficits may constitute an endophenotype of bipolar disorder (Bora et al., 2009). Given that relatives of bipolar patients showed signs that they exhibited some mild symptomatology—evidenced by elevations in SPQ scores—greater reaction times in this group may reflect impaired concept shifting (Arts et al., 2008). Likewise, relatives of patients with schizophrenia showed similar signs of subtle symptomatology. In this context, greater SimDiff errors in these relatives partially supports the hypothesis that impaired contour detection is associated with genetic liability for schizophrenia.

## 5 Limitations

The present study has limitations that warrant consideration. The imbalance between number of male and female probands complicates the observed interactions between diagnostic group and gender: unequal sample sizes across categorical subgroups inflates the Type I Error Rate (Aguinis et al., 1999). Given that all observed statistical interactions between diagnostic group and gender in the present study have a smaller partial η^2^ than the main effect of group alone suggests that diagnostic group differences appear to better account for observed differences in behavior and neural functions than the interactive effects between group and gender. Likewise, we acknowledge that the candidate gene concept in schizophrenia has been largely replaced by genome wide association studies—the former has largely failed to yield insights into the genetic basis of schizophrenia (Collins et al., 2012, Farrell et al., 2015). Critically, COMT polymorphisms appear to be reliably associated with differential higher order processing (Mier et al., 2010), and appear to be a valid marker of dopaminergic function relative to other candidate genes (Tunbridge et al., 2019). In this context, our present findings of differential effects of COMT variation on neural functions related to higher-order processing in individuals with liability for schizophrenia and bipolar disorder are interesting, though must be interpreted with caution.

The large number of schizophrenic probands with a history of alcohol dependence could possibly confound the observed reductions in P3b amplitudes relative to healthy controls in the present study. Alcohol dependence is associated with reduced P3b amplitude (Hamidovic and Wang, 2019). In addition, it appears to mark high-risk status for developing alcoholism (Hill and Shen, 2002), and persists after sustained periods of abstinence (Fein and Chang, 2006). In order to account for the possible confounding effects of alcohol dependence, a Hotelling’s T^2^ test examining P3b amplitudes at P7 and P8 during vigilance and “press every trials” compared healthy controls and patients with schizophrenia, using history of alcohol dependence as a categorical covariate. Patients with schizophrenia display reduced P3b components relative to healthy controls (*T^2^*(1 0 8) = 4.00, *p* < .01), and there was no effect of history of alcohol dependence (*T^2^*(1 0 8) = 0.015, *p* = . 80), suggesting that a history of alcohol dependence does not account for observed group differences in the present sample.

## 6 Conclusions

The present study documents differential neurophysiological responses to the DS-CPT in individuals with liability for schizophrenia and bipolar disorder. Both relative groups displayed modulated N1 components, though this modulation appears compensatory only in relatives of patients with schizophrenia. Coupled with a larger number of false positive errors to stimuli with contours shared with target stimuli suggests impaired contour detection may reflect the neural consequences of genetic liability for schizophrenia. Deficits in N2 difference waveforms in patients with schizophrenia suggests a disorder specific abnormality related to object recognition. Diminished P3b amplitudes may constitute an endophenotype for schizophrenia building on our previous findings (Sponheim et al., 2006), and suggests that deficits in neural functions implicated in ventral attentional and salience networks during visual target detection may mark genetic liability for schizophrenia, but not bipolar disorder (Wynn et al., 2015). Finally, we provide novel evidence that COMT variation differentially impacts neural functions in individuals with genetic liability for schizophrenia versus bipolar disorder. Collectively, our findings suggest that aberrant neural responses and not performance on the DS-CPT better differentiate liability for schizophrenia from bipolar disorder, and that fronto-parietal dysfunction related to classifying salient features of visual stimuli may serve as an endophenotype specific to schizophrenia.

## Authors contribution

Conceptualization was provided by Scott R Sponheim and Samuel Klein. Administration, investigation, and methodology was provided by Laurie Shekels, Kathryn McGuire and Scott Sponheim. Funding was procured by Scott R Sponheim. Validation, software visualization and formal analysis was completed by Samuel Klein. Finally, Samuel Klein wrote the manuscript, with Scott Sponheim substantially editing the manuscript. Laurie Shekels and Kathryn reviewed the manuscript, and also provided edits.

## Declaration of Competing Interest

The authors declare that they have no known competing financial interests or personal relationships that could have appeared to influence the work reported in this paper.
