# Supplementary material for: Neural anomalies during vigilance in schizophrenia: Diagnostic specificity and genetic associations
Source: Neuroimage Clin. 2020 Sep 8;28:102414. doi: 10.1016/j.nicl.2020.102414 (PMC7502576; doi:10.1016/j.nicl.2020.102414)

**Methods**

**COMT Data Processing**

We determined the Val^158^Met COMT genotype for each individual by implementing a restriction fragment length polymorphism technique. Whole blood was collected on FTA Matrix specimen collection cards (Whatman). Subsequently, punches from the FTA blood cards were prepared for polymerase chain reaction (PCR) analysis according to Whatman FTA protocol. The washed punch was then used for PCR amplification. Primers for PCR amplification spanned the COMT Val^158^Met polymorphism (SNP rs4680) (forward primer 5′ tactgtggctactcagctgtgc 3′, reverse primer 5′ gtgaacgtggtgtgaacacc 3′). Initially, PCRs were denatured at 94°C for 3 min followed by 39 cycles of denaturation at 93°C for 45 s, annealing at 55°C for 1 min and extension at 72°C for 1 min with a final 4 min extension at 72°C. The PCR products were digested with NlaIII (New England Biolabs, Ipswich, MA) for 3 h at 37°C followed by incubation at 60°C for 20 min to denature the enzyme. The digestion was then separated by polyacrylamide gel electrophoresis and the digestion products visualized by staining with ethidium bromide. The COMT val allele has a G at position 1947, yielding 114 base pair fragments after digestion with NlaIII. In contrast, the COMT Met allele has an A at this position which allows digestion of the 114 base pair fragment into 2 products of 96 and 18 base pairs.

**Contrasts of ERPs Across All Five Groups**

**Early Posterior Potential: N1**

A MANOVA examining the N1 component in HC, SZ, SZRel, BP and BPRel at O1 and O2 revealed a main effect of task (*F*_(_*_1,208)_* = 21.76, *p* <.001, Wilk’s Λ =.908, partial η^2^ = .092), where N1 was greater during vigilance (M = -5.16, SE =..382) than sensory control trials (M = -4.12, SE = .376; Sidak corrected *p* < .001). There was also an interaction between task, group and gender (*F*_(_*_4,208)_* = 4., *p* = .048, Wilk’s Λ =.955, partial η^2^ = .045). There was no effect of gender (F_(1,208)_ = .918, p = .34, η^2^ = .009), though there was a main effect of group (F_(4,208)_ = 2.94, p = .02, η^2^ = .054), with no observed interaction between group and gender (F_(4,208)_ = .811, p = .468, η^2^ = .009). Post-Hoc comparisons revealed that SZRel had greater N1 amplitudes than HC (FDR corrected *p* = .04), SZ (FDR corrected *p* = .02), and BPAD (FDR corrected *p* = .02), while BRel had greater N1 than BPAD (FDR corrected *p* = .03). A main effect of hemisphere was also observed (*F*_(_*_1,208)_* = 6.77, *p* = .01, Wilk’s Λ =.987, partial η^2^ = .04) such that amplitudes were greater at O2 (i.e. right hemisphere; M = -4.86, SE = .42) than at O1 (M = -4.68, SE =.39; Sidak corrected *p* = .01). There were interactions between task and target (*F*_(_*_1,208)_* = 5.48, *p* = < .01, Wilk’s Λ =.966, partial η^2^ = .034), with follow-up simple main-effects analyses revealing that N1 amplitudes were greater to target (Sidak corrected *p* < .001) and nontarget trials (Sidak corrected *p* = .036).

**Middle Latency Potential: N2**

A MANOVA examining group differences between HC, SZ, SZRel, BP and BPRel revealed a main effect of group (F_(4,212)_ = 3.15, p < .015, η^2^ = .056) with follow-up post-hocs revealing that SZ had reduced amplitudes compared to HC (FDR corrected *p* = .01). There was a main effect of gender (F_(1,212)_ = 2.85, p = .02, η^2^ = .017) with males having greater amplitudes (M = -.63, SE = .27) than females (M= .482, SE =.391, *p* = .02), and no observed interaction between gender and group (F_(4,212)_ = .327, p =.86, η^2^ = .003). There were interactions between electrode site and group (*F*_(_*_2, 212)_* = 3.25 , *p* = .013 , Wilk’s Λ =.942, partial η^2^ = .058). An effect of electrode (*F*_(_*_1,212)_* = 85.04, *p* <.001 , Wilk’s Λ =.714, partial η^2^ = .286) reflected that N2 difference waveforms were greater at Cz (M = -8.14, SE = .308) than Fz (M = .595, SE = .246; Sidak corrected *p <* .001). Follow-up analyses for each electrode site revealed that SZ had reduced N2 difference waveforms at Fz (M = 1.31, SE = .645) compared to HC (M = -.24, SE = .33; Sidak corrected *p* < .01).

**Posterior Potential: P3b**

A MANOVA examining the P3b component to targets at electrode sites P7 and P8 in HC, SZ, SZRel, BP and BPRel revealed a significant effect of task (*F*_(_*_1,209)_* = 160.26, *p* <.001, Wilk’s Λ =.564, partial η^2^ = .435). Amplitudes were greater during vigilance (M = 6.97, SE = .265) relative to “press every” control trials (M =3.29, SE = .16; Sidak corrected *p* < .001). There were also main effects of group (*F_(2,162)_* = 3.14, *p* =.015, partial η^2^ = .05), gender (*F_(1,162)_* = 6.85, *p* =.01, partial η^2^ = .057), and an interaction between gender and group (*F_(2,162)_* = 3.161, *p* =.045, partial η^2^ = .032). Post-hoc comparisons revealed that SZ, BPRel and SZRel had smaller P3bs than HC (FDR corrected *p <* .001*;* FDR corrected *p =* .037; and FDR corrected *p* = .024 respectively). There was also an interaction between task and side, with follow-up simple main effects revealing that amplitudes were greater at P8 (M= 3.52, SE = .18) than P7 (M = 3.06, SE =.182, *p* < .01).

**COMT and Liability for Schizophrenia (N2)**

A MANOVA examining how N2 difference waveforms differed by genotype in HC, SZ and SRel, and genotype revealed a significant effect of region (*F*_(_*_1,115)_* = 72.51, *p* <.001, Wilk’s Λ =.613, partial η^2^ = .387), with amplitudes being larger at Cz (M=-1.373, SE =.36) than Fz (M =.22, SE =.27; Sidak corrected *p* < .001). There was an interaction between region and group code (*F*_(_*_1,115)_* = 9.33, *p* <.001, Wilk’s Λ =.86, partial η^2^ = .14), with follow up simple main effect analyses showing that HC had reduced N2 amplitudes at Fz (M= -1.07, SE =.544) relative to SZ (M =1.03, SE =.41; Sidak corrected *p* <.01) and relatives of schizophrenia probands (M = .709, SE = .41; Sidak corrected *p* = .012). Similarly, HC had greater N2 amplitudes at Cz (M = -3.3, SE =.75) than schizophrenia probands (M = .51, SE =.54; Sidak corrected *p* <.001) and first-degree relatives of schizophrenia probands (M =-1.32, SE =.54; Sidak corrected *p* = .036). There was also a main effect of group (*F_(2,115)_* = 7.07, *p* < .01, partial η^2^ = .109), with follow up post hoc tests revealing that HC had greater amplitudes compared to both schizophrenia probands (Tukey’s HSD *p* < .001) and first-degree relatives of probands (Tukey’s HSD *p* < .01). There was no effect of genotype group (*F_(1,115)_* = .125, *p* = .883, partial η^2^ = .002) and no interaction between group and genotype (*F_(4,115)_* = ..881, *p* = .478, partial η^2^ = .030).

**COMT Summary**

Collapsed across HC, SZ and their first-degree relatives, val homozygotes had reduced P3b waveforms relative to val/met heterozygotes and met homozygotes during vigilance. In contrast, when examining COMT effects in HC, patients with bipolar disorder and their first degree relaitves, val/met heterozygotes had the largest reduction in P3b waveforms relative to met homozygotes, with val homozygotes having intermediate amplitudes. Collapsing across schizophrenic probands and their relatives, COMT genotype group revealed differential positive associations between neural functions and perceptual sensitivity: N1 amplitude in met homozygotes, N2 difference waveforms amplitudes in val/met heterozygotes, and P3b amplitude in val homozygotes. In val/val patients, chlorpromazine equivalence was positively associated with P3b amplitudes.

*Supplementary Table 2.* Proband Comparisons

|  | Schizophrenia Probands (2006 Sample) *N = 22* | Newly Recruited Probands (added to present work)  *N =26* | Test Statistic | *p* value |
| --- | --- | --- | --- | --- |
| Target Detection: d’ | 2.10 (.71) | 2.68 (1.03)^a^ | *t(46) = 2.22* | 0.03 |
| Cpz Equivalents | 628 (436) | 768 (643) | *t(46) =0.87* | .39 |
| Estimated IQ | 98.2 (9.6) | 96.5 (12.5) | *t(46) = -.53* | .60 |
| Years of Education | 14.2 (2.8) | 13.7 (2.9) | *t(46) = -.65* | .52 |
| BRELS Total | 40.6 (10.8) | 43.1 (11.4) | *t(46) = -.65* | .52 |
| SAPS Total | 17.0 (14.5 | 14.4 (13.4) | *t(46) = -1.26* | .21 |
| SANS Total | 24.5 (11.5) | 23.2 (13.1) | *t(46)= -.04* | .97 |
| Years of Education | 14.2 (2.25) | 13.7 (2.89) | *t(46)= -.65* | .52 |
| Visual Acuity (LogMAR) | .23 (.34) | .36 (.41) | *t(46)= 1.04* | .31 |
|  |  |  |  |  |

^a^ Different from 2006 Sample mean, *p* < .05

*Supplementary Table 3.* Bipolar Proband Comparisons

|  | Bipolar Probands (without history of psychotic features)  *N =16* | | Bipolar Probands (with history of psychotic features)  *N =10* | Test Statistic | *p* value |
| --- | --- | --- | --- | --- | --- |
| Target Detection: d’ | 2.30 (1.04) | | 2.48 (.91) | *t(24) =.452* | 0.65 |
| N1 Amplitude | **O1:** -3.37 (1.09)  **O2:** -3.77 (1.14) | | **O1:** -3.84 (1.38)  **O2:** -4.16 (1.44) | *T^2^(23) = .04* | .961 |
| N2 Difference Amplitude | **Cz:** -.516 (.76)  **Fz:**  .962 (.64) | | **Cz:** -2.09 (.96)  **Fz:** .451 (.81) | *T^2^(23) = 1.81* | .187 |
| P3b Amplitude | **P7:** 6.57 (.96)  **P8:** 6.39 (.89) | | **P7:** 7.07 (.96)  **P8:** 7.31 (1.1) | *T^2^(23) = .40* | .675 |
| *T^2^*: Hotelling’s T^2^ test (multivariate t-test | |  |  |  |  |

**Supplementary Figure 1**


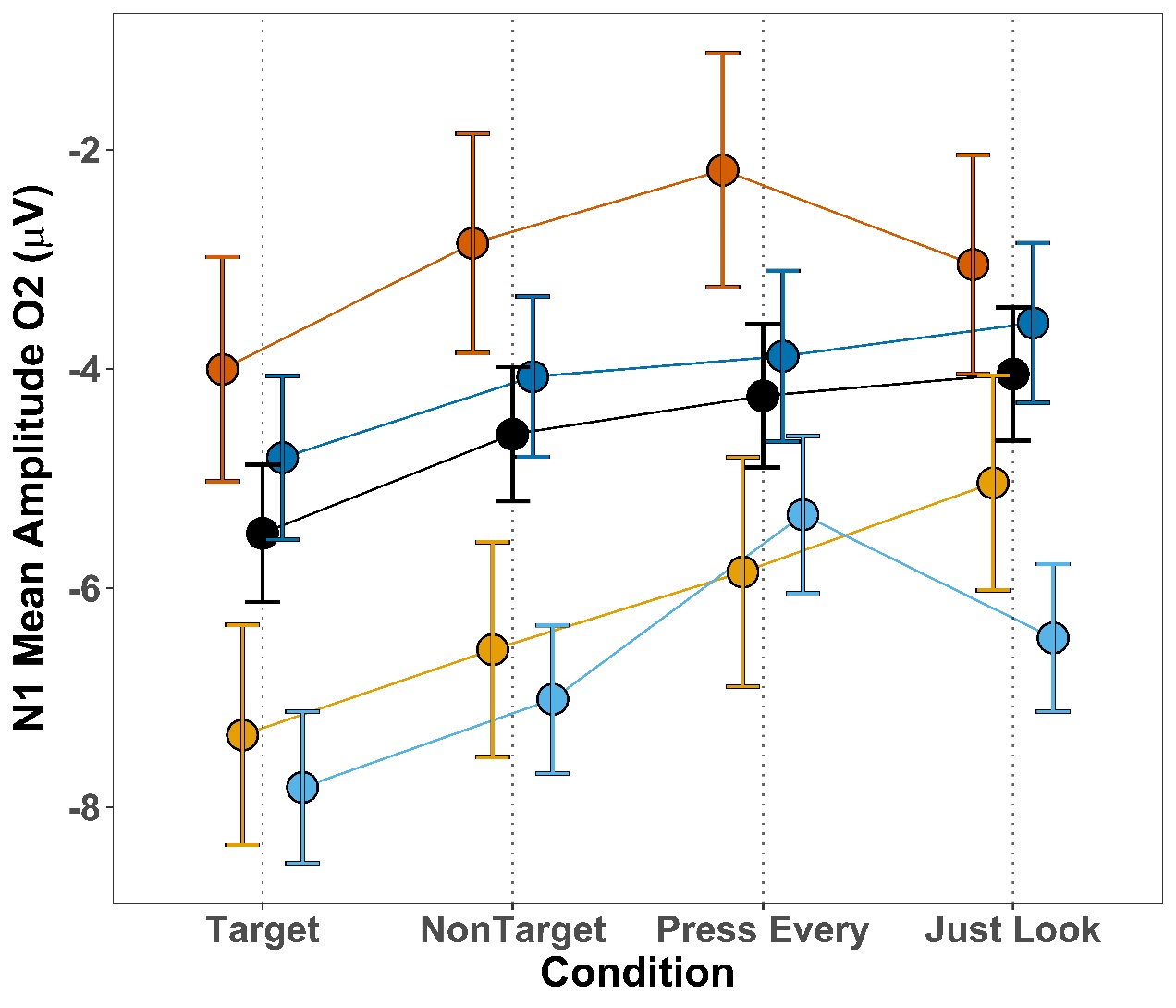

Supplement: Supplementary data 1 [file mmc1.docx]
